# Drinking Water from Dug Wells in Rural Ghana — *Salmonella* Contamination, Environmental Factors, and Genotypes

**DOI:** 10.3390/ijerph120403535

**Published:** 2015-03-27

**Authors:** Denise Myriam Dekker, Ralf Krumkamp, Nimako Sarpong, Hagen Frickmann, Kennedy Gyau Boahen, Michael Frimpong, Renate Asare, Richard Larbi, Ralf Matthias Hagen, Sven Poppert, Wolfgang Rabsch, Florian Marks, Yaw Adu-Sarkodie, Jürgen May

**Affiliations:** 1 Research Group Infectious Disease Epidemiology, Bernhard Nocht Institute for Tropical Medicine, Bernhard-Nocht-Straße 74, Hamburg 20359, Germany; E-Mails: krumkamp@bnitm.de (R.K.); may@bnitm.de (J.M.); 2 German Centre for Infection Research (DZIF), Hamburg-Borstel-Lübeck, Bernhard-Nocht-Straße 74, Hamburg 20359, Germany; 3 Kumasi Centre for Collaborative Research, Kumasi, Ghana; E-Mails: nimakosarpong@yahoo.com (N.S.); gyaukennedy@yahoo.com (K.G.B.); mfrimpong28@gmail.com (M.F.); browny2gh@yahoo.com (R.A.); kerl18mcc@hotmail.com (R.L.); 4 Department of Tropical Medicine, German Armed Forces Hospital of Hamburg, Bernhard Nocht Institute for Tropical Medicine, Bernhard-Nocht-Straße 74, Hamburg 20359, Germany; E-Mails: frickmann@bnitm.de (H.F.); hagen@bnitm.de (R.M.H.); 5 Institute for Medical Microbiology, Virology and Hygiene, University Hospital Rostock, Schillingallee 70, Rostock 18057, Germany; 6 Justus-Liebig-University Gießen, Rudolf-Buchheimstraße 6, Gießen 35392, Germany; E-Mail: sven@poppert.eu; 7 Robert Koch Institute, FG11, National Reference Centre for *Salmonella* and other Bacterial Enteric Pathogens, Burgstraße 37, Wernigerode 38855, Germany; E-Mail: rabschw@rki.de; 8 International Vaccine Institute, 1 Gwanak-ro, Gwanak-gu, Seoul 151-742, South Korea; E-Mail: fmarks@ivi.int; 9 Kwame Nkrumah University of Science and Technology (KNUST), Accra Road, Kumasi, Ghana; E-Mail: yasax@hotmail.co.uk

**Keywords:** *Salmonella*, disease transmission, drinking water, dug wells, risk factor

## Abstract

Salmonellosis is an important but neglected disease in sub-Saharan Africa. Food or fecal-oral associated transmissions are the primary cause of infections, while the role of waterborne transmission is unclear. Samples were collected from different dug wells in a rural area of Ghana and analyzed for contamination with bacteria, and with *Salmonella* in particular. In addition, temporal dynamics and riks factors for contamination were investigated in 16 wells. For all *Salmonella* isolates antibiotic susceptibility testing was performed, serovars were determined and strains from the same well with the same serovar were genotyped. The frequency of well water contamination with Gram-negative rod-shaped bacteria was 99.2% (n = 395). Out of 398 samples, 26 (6.5%) tested positive for *Salmonella* spp. The serovar distribution was diverse including strains not commonly isolated from clinical samples. Resistance to locally applied antibiotics or resistance to fluoroquinolones was not seen in the *Salmonella* isolates. The risk of *Salmonella* contamination was lower in wells surrounded by a frame and higher during the rainy season. The study confirms the overall poor microbiological quality of well water in a resource-poor area of Ghana. Well contamination with *Salmonella* poses a potential threat of infection, thus highlighting the important role of drinking water safety in infectious disease control.

## 1. Introduction

Non-typhoid *Salmonella* (NTS) are distributed worldwide, in industrialized, as well as in resource-limited countries. The course of NTS infection in industrialized countries is usually a self-limiting diarrheal disease, and bloodstream infections are rare, occurring mainly in immunocompromised individuals [1]. In contrast, in sub-Saharan Africa, NTS are among the most common causes of bacteraemia [2,3]. In Ghana, studies demonstrated that *Salmonella enterica* is one of the most frequent blood culture isolates in febrile children [4,5,6].

Humans get infected with *Salmonella* through the consumption of contaminated food of animal origin, such as poultry, egg and milk products, and contaminated water, or via the fecal-oral route. In Africa, access to bottled drinking water especially in rural areas is rare and the role of drinking water in *Salmonella* infection is unclear. Main sources for drinking water are rivers, lakes and wells, which often are contaminated by soil, rubbish, dust, and animal droppings. Previous studies in Africa have emphasized the problem of drinking water contamination not only with fecal bacteria but also with *Salmonella*, indicating that such water may be unsafe for consumption [7,8]. In Ghana, little information is available on the prevalence of *Salmonella* serovars in water sources and whether these are associated with human infections. In addition, there is no data describing the association of well characteristics and other risk factors for well water contamination with *Salmonella*.

Supply of safe drinking water is high on the political agenda of international institutions. The Millennium Development Goal (MDG) 7.C, released in 2000 by the United Nations, aspires to halve the proportion of people without sustainable access to safe drinking water and basic sanitation by the end of 2015 [9]. The World Health Organization (WHO) has been regularly updating their guidelines for drinking water quality [10]. Even though much has been learned in recent years, data on risk factors for water and sanitation related infections and modes of water-borne pathogen transmission are scarce, but needed to guide local prevention programs. Hence, to assess drinking water quality, water sources have to be assessed for fecal contamination. To understand whether the same *Salmonella* strains present in water sources are associated with human infections, typing of *Salmonella* isolates found in drinking water sources is necessary. The question, how to protect a water source from fecal contamination seems to be simple, however, in order to protect the health of water users it is important to identify simple measures, such as putting a lid on the well, which may reduce contamination of water by enteric pathogens. The aim of this study was to generate information on (i) the frequency of water contamination, in particular with *Salmonella* *enterica*, in wells over time and (ii) to determine external factors associated with well water contamination in order to recommend on contamination preventing well construction and maintenance. The study was conducted in a rural village within the Asante Akyem District in Ghana, an area where many people use local dug wells as drinking water sources. 

## 2. Methods

In 2009 and 2010, well water samples were collected in Asankare, a rural village situated in the Asante Akyem District in Ghana. Asankare is a typical rural village of Ghana, in which improved sanitation facilities are not available. Livestock is commonly found in the area of the wells and surface water from rivers and lakes is present. In the rainy seasons, flooding of the area is frequent. 

**Figure 1:**
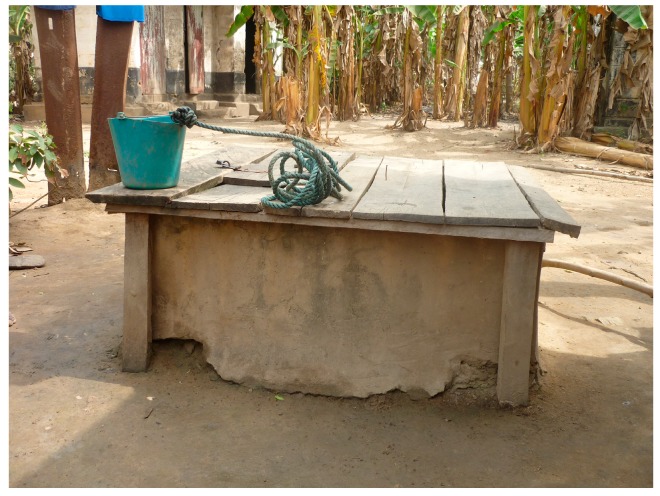
Example of a covered well surrounded by a frame in the village of Asankare, Asante Akyem District, Ghana (copyright Denise Dekker).

Different sources for drinking water existed, however the main water sources were local dug wells (Figure 1). Sixteen wells with guaranteed access throughout the study period were randomly selected. These wells were sampled once every two weeks for a period of one year and temporal changes of contamination investigated. In total, wells were sampled 26-times throughout the study year. 

For each sample, 200 mL water was taken from a well, 100 mL for culturing Gram-negative rod-shaped bacteria and 100 mL for culturing *Salmonella*. Samples were transported to the microbiology laboratory of the Kumasi Centre for Collaborative Research in Tropical Medicine (KCCR) in a cool box containing ice packs within 4 hours after sample collection. At KCCR, each sub-sample was filtered using a 0.45 μm pore cellulose membrane filter (Millipore, Cork, Ireland). For the colony counts of Gram-negative rods, the filter was directly placed onto a MacConkey agar selective for Gram- negative bacteria (Oxoid Ltd., Basingstoke, Hampshire, England) and incubated at 35–37 °C for 18–24 h. Following incubation, the colony counts for total Gram-negatives were classified into three groups, *i.e.*, <10, 10–100 or >100 colony forming units (CFU) per 100 mL. 

To culture *Salmonella*, the filter was placed in Selenite F broth (Oxoid) for enrichment, the broth streaked after 18–24 h incubation on a chromogenic selective *Salmonella* agar (Oxoid) and incubated at 35–37 °C for 18–24 h. From positive *Salmonella* cultures, one pure colony was picked and used for further testing. *Salmonella* were identified using a latex agglutination Test (Oxoid) and the API 20E biochemical identification strip (bioMérieux, Marcy L’etoile, France). Susceptibility was tested for a set of antibiotics commonly used in the study area, *i.e.*, ampicillin, ampicillin/sulbactam, ceftriaxone, chloramphenicol, ciprofloxacin, cotrimoxazole, nalidixic acid (used as a screening test for ciprofloxacin) and tetracycline, following the Clinical and Laboratory Standards Institute (CLSI) guidelines. *Salmonella* isolates were stored at –80 °C until transport to Germany on dry ice. For all *Salmonella* isolates, the serovar was determined by slide agglutination following the Max von Gruber method [11] using *Salmonella* specific antisera (SIFIN GmbH, Berlin, Germany) and the White-Kauffmann-Le Minor Scheme [12] (carried out at the Bernhard Nocht Institute for Tropical Medicine). 

To establish relationships amongst the isolated *Salmonella* from water samples, strains with the same serovar and from the same well were further typed by Pulsed-field gel electrophoresis (PFGE). After *XbaI* (Roche Diagnostics GmbH, Mannheim, Germany) restriction, PFGE settings were as follows: initial switch time 1 minute, final switch time 40 seconds, run time 22 hours, voltage 6 V/cm and included angle 120°. Gel Compar II software (Applied Maths, Sint-Martens-Latem, Belgium) was used to compare PFGE patterns, using DICE, UPGMA, 9.5% optimization, 1.0% position tolerance, with Lambda Ladder as a molecular marker (carried out at the Institute for Medical Microbiology, Virology and Hygiene, University Hospital Rostock).

For each well, contamination was assessed using the frequency with which CFU Gram-negative rod-shaped bacteria were found in water samples and the number of positive *Salmonella* cultures observed throughout the study year.

Furthermore, repeated water contamination in wells was analyzed. When a well was repeatedly contaminated with *Salmonella*, the serovar identified in each of these samples along with its clonal identity were assessed to determine *Salmonella* persistence.

The longitudinal data of the repeatedly sampled wells was analyzed to establish the link between *Salmonella* contamination, well characteristics, and seasonality. The dataset follows a hierarchical structure, where water samples are taken at different time points (level one-unit) from particular wells (level two-unit). Hence, a random-effect regression model for repeated binary responses was applied to analyze the data [13]. Sampling points at which identical *Salmonella* clones were identified in consecutive water samples were removed from the analysis, as persistent contamination was assumed. Missing data, e.g., when a well was locked or dried out, were excluded from the analysis. The following covariates were considered to describe well characteristics: closure of well (wooden/ metal cover), presence of a frame (concrete frame, categorized into below or above 30 cm), presence of rubbish in well and seasonality (rainy *vs.* dry season). Odds Ratios (OR) and the corresponding 95% confidence intervals (CI) were calculated. Adjusted ORs are reported from multivariate regression models to account for confounding. Model selection was based on a likelihood-ratio test. 

## 3. Results 

Sixteen wells were sampled biweekly from 10 November 2009 to 17 November 2010, *i.e.*, at 26 sampling time-points. Eighteen samples could not be collected; hence 398 water samples were analyzed. The analysis is divided into the following parts: (i) well contamination with Gram-negative rod-shaped bacteria and *Salmonella*, (ii) *Salmonella* serovars and antibiotic susceptibility, (iii) PFGE genotypes of consecutively contaminated wells, and (iv) well characteristics and their associations with *Salmonella* contamination. 

### 3.1. Contamination with Gram-Negative Rod-Shaped Bacteria 

Out of the 398 water samples tested, 395 (99.2%) were contaminated with >100 CFU/100 mL Gram-negative rods. In the remaining three (0.8%) water samples between 10 and 100 CFU/100 mL were detected. 

### 3.2. Salmonella Isolates and Serovars

**Figure 2:**
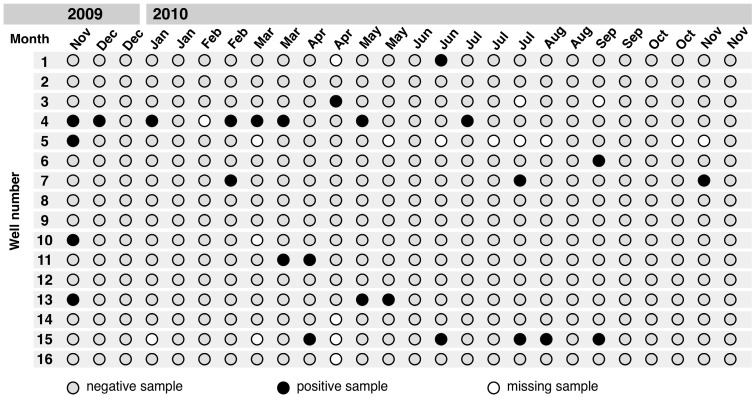
*Salmonella* contamination in 16 dug wells throughout the study period in Asankare, Asante Akyem District, November 2009–November 2010.

Of the 398 tested well water samples, 26 (6.5%) tested positive for *Salmonella* (Figure 2). One isolate was lost during culture for serotyping and in one sample two serovars were detected. The following serovars were identified: *S.* Pramiso (n = 2), *S*. Stanleyville (n = 4), *S*. Rubislaw (n = 4), *S*. Duisburg (n = 3), *S*. Nima (n = 2), *S*. Saarbruecken (n = 3), *S*. Give (n = 1) and *S*. Colindale (n = 7). *Salmonella* were never isolated from six of the 16 study wells. The remaining ten wells tested positive between one and eight times each. 

### 3.3. Clusters of Salmonella Isolates

Contamination of wells with *Salmonella* was highest in November 2009. *Salmonella* clusters were observed from February 2010 until May 2010. *Salmonella* colonization was less frequent from January 2010 until February 2010, and from September 2010 until November 2010. Seasonal associations and *Salmonella* clusters are further described below. 

### 3.4. Antibiotic Susceptibility 

Antibiotic susceptibility of *Salmonella* is shown in Table 1. Resistance was restricted to ampicillin and tetracycline in one of the isolates and to ampicillin and ampicillin/sulbactam in another isolate.

**Table 1:** Antibiotic susceptibility of *Salmonella* isolates (N = 26) found in Asankare wells, Asante Akyem District, November 2009–November 2010.

	Frequency (%)	
Drug (AC μg)	Susceptible	Resistant
Ampicillin (10)	24 (92.3)	2 (7.7)
Ampicillin/Sulbactam (20)	25 (96.2)	1 (3.8)
Ceftriaxone (30)	26 (100.0)	0 (0)
Chloramphenicol (30)	26 (100.0)	0 (0)
Ciprofloxacin (5)	26 (100.0)	0 (0)
Cotrimoxazole (25)	26 (100.0)	0 (0)
Nalidixic acid (30)	26 (100.0)	0 (0)
Tetracycline (30)	25 (96.2)	1 (3.8)

AC: antibiotic concentration.

### 3.5. PFGE Genotyping 

**Figure 3:**
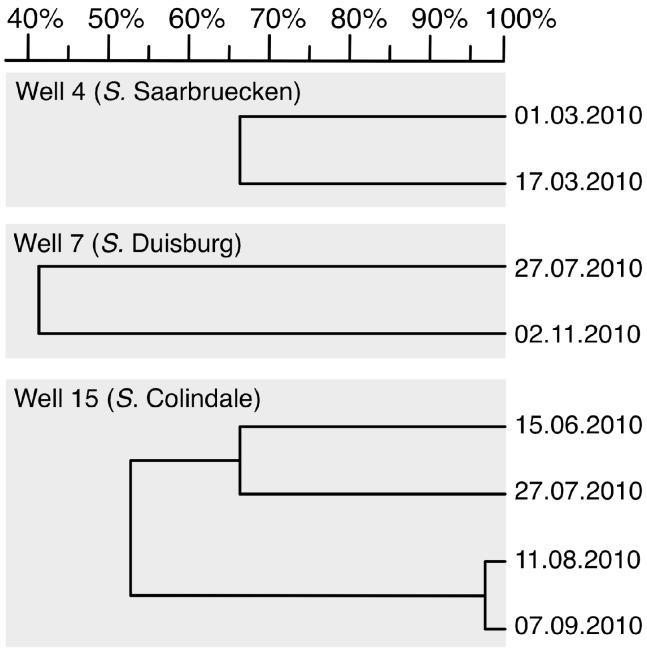
Dendrogram of pulsed-field gel electrophoresis (PFGE) profiles of three different serovars showing the genetic relationships for *Salmonella* isolates in Asankare wells, Asante Akyem District. The numbers on the right indicate sampling date. The numbers on the top (scale) indicate the genetic identity in percent.

*Salmonella* of the same serovar and isolated from the same well were eligible for PFGE to identify clonal clusters. Clonal identity was accepted at 95 % identity. In total, eight *Salmonella* isolates from three wells were discriminated to seven distinct clones by PFGE. Thus, individual wells were mostly colonized by different strains. From one well, a single clone persisted for about four weeks. PFGE results stratified by well are shown in Figure 3. 

### 3.6. Well Characteristics and Salmonella Contamination 

Three hundred and ninety-seven well water samples were included in this analysis, of which 25 (6.3%) were *Salmonella* positive and 372 (93.7%) were negative. One observation was discarded because of persistent *Salmonella* contamination. Most samples were from covered wells (n = 269; 67.8%) and from wells with a frame (n = 294; 74.1%). The majority of samples were collected during the rainy seasons, from March to July, and September to November. Table 2 shows bivariate (Model A–D) and multivariate (Model E) random-effect models on well characteristics and their associations with *Salmonella* contamination. In the bivariate models, a frame with a minimum height of 30 cm indicated a possible protective association (OR: 0.3; 95% CI = 0.1–1.3) and rainy season a risk factor (OR: 2.6; 95% CI = 1.2–5.6) for *Salmonella* contamination. The multivariate model confirmed the protective effect of a well frame (OR: 0.3; 95% CI = 0.1–0.8) and the risk of rainy season (OR: 2.6; 95% CI = 1.2–5.5). In addition, an association between *Salmonella* contamination and the presence of rubbish was indicated (OR: 2.9; 95% CI = 0.9–9.6). 

**Table 2:** Description of well characteristics and results from the random-effect models for well characteristics and *Salmonella* contamination, Asankare wells (397 sampling occasions from 16 wells), Asante Akyem District.

Independent Variables	Frequency (%)	Random-Effect Models OR (95% CI)				
		Model A	Model B	Model C	Model D	Model E
Presence of frame (>30 cm)	294 (74.1)	0.3 (0.1–1.3)	–	–	–	0.3 (0.1–0.8)
Well covered	269 (67.8)	–	2.0 (0.3–13.6)	–	–	–
Rubbish in well	75 (18.9)	–	–	2.6 (0.9–7.4)	–	2.9 (0.9–9.6)
Season (rainy *versus* dry)	272 (68.5)	–	–	–	2.6 (1.2–5.6)	2.6 (1.2–5.5)
Random Intercept Variance (SE)	–	0.89 (0.82)	2.96 (3.21)	0.96 (0.49)	1.68 (0.97)	0.76 (1.28)

OR: odds ratio; aOR: adjusted odds ratio; CI: confidence interval; SE: standard error.

## 4. Discussion

The main findings of this study are that (i) the majority of well water samples (99.2%) were contaminated with Gram-negative rod-shaped bacteria with >100 CFU/100 mL, (ii) 6.5% of water samples were contaminated with *Salmonella*, (iii) resistance to locally administered antibiotics was negligible in the *Salmonella* strains isolated from well water, (iv) the serovar distribution consisted of serovars not typically seen in clinical specimens, (v) wells were colonized by strains that persist for several weeks and (vi) certain well characteristics were associated with *Salmonella* contamination.

WHO standards for potable water demand the total absence of coliforms and fecal indicator bacteria [14]. In this study, nearly all samples were contaminated with Gram-negative rod-shaped bacteria above 100 CFU/100 mL, indicating a potential health hazard for the local population. The current study could not reveal the spectrum of organisms present in the water samples, nevertheless the colonial morphology pointed to fecal contamination. The number of infections actually transmitted via well water was not assessed since simultaneous gastrointestinal infections were not monitored during the study period. 

At present, little information is available on the prevalence of *Salmonella* serovars in water sources in developing countries, yet studies have shown the presence of various human-pathogenic organisms in the aquatic environment [15,16,17]. Despite these reports, the *Salmonella* serovars identified in the present study are not those typically seen in clinical samples, but are rather known to be associated with reptiles or poultry. Serovars commonly found in reptiles are *S.* Pramiso [18], *S.* Rubislaw [19], and *S.* Nima [20]. *S.* Duisburg [21] and *S.* Saarbruecken [22] are found in poultry. Isolated serovars with pathogenic potential for humans are *S.* Stanleyville [23], *S.* Give [24] and *S.* Colindale [25]. Nevertheless, it has to be considered that all detected pathogens occur infrequently in clinical samples. 

In the aquatic environment *Salmonella* are expected to be present in low concentrations. As only quite small amounts of water were sampled, the test sensitivity may be limited. In addition, the reported number of serovars per sample may be an underestimate as per positive sample only one colony was picked for further testing and, thus, for serotyping.

PFGE genotyping showed identical *Salmonella* clones at different sampling time points. This could be a result of repeated contaminations with the same genotype circulating in the environment but is more likely due to persistence of distinct clones for several weeks. Previous work has shown the capability of *Salmonella* to survive in water for several months [26]. 

Our study demonstrates well characteristics associated with *Salmonella* contamination. A frame around the well is likely to have a protective effect as it serves as a barrier against intrusion by animals, soil and dust. This effect was observed in a previous study on well water quality, where lined wells topped with a concrete parapet had lower concentrations of fecal indicator bacteria, especially at the beginning of the wet season [27]. A frame might prevent contamination of well water by surface run offs containing animal and human feces and soil, an assumption supported by the observed association between *Salmonella* contamination and rainy season. In rural areas, like in the Asankare village, animals roam freely. Studies in stray dogs and cats in the United States have shown carriages of *Salmonella* as high as 51.4% [28]. Reptiles and amphibians, that can easily fall or hide inside the wells, are also potential *Salmonella* carriers [29]. An additional risk factor for *Salmonella* contamination may be the presence of rubbish in well water. Rubbish might just indicate that the well is prone to contamination generally or it may contribute directly to *Salmonella* contamination. 

Limitations of this research include the fact that the calculated odds ratios between environmental factors and *Salmonella* presence are of low precision. Furthermore, the assessed risk factors were not studied on a larger scale. For instance no data on the weekly precipitation was available and the frame height was analyzed without considering construction quality. Studying these associations with a larger sample size throughout a broader study region and including other potential risk factors would generate more valuable information to guide well construction and maintenance. In addition, the culture results of the Gram-negative rod-shaped bacteria were not reported as CFU per 100 ml but rather as categories, hence nearly all data fit a single category. As a consequence, it is not possible to identify measures of Gram-negative rods that might indicate relatively low probability of contamination.

It is estimated that 10% of the total burden of disease worldwide is related to water, sanitation and hygiene. Of this 62% are associated with drinking water and sanitation alone [30], mainly childhood diarrheal diseases. To evaluate progress towards the MDGs water targets, water sources are classified as improved drinking water sources (*i.e.*, piped water on premises, public taps, boreholes, or protected dug wells) or unimproved sources (*i.e.*, unprotected dug wells, unprotected springs or surface water) [31]. Globally, access to improved water sources has increased during recent years and the MDGs drinking water target has become one of the first Millennium Goals to be met [32]. However, significant disparities exist between and within regions, for example access to improved water sources is lowest in sub-Saharan Africa and in the poorer rural areas within countries [14]. In the current study, all wells were contaminated at nearly all sampling dates with fecal indicator organisms above the WHO threshold. Interestingly, the sampled wells belong to both categories, protected and unprotected dug wells. The current study suggests a more sophisticated classification may be necessary to differentiate between improved and unimproved sources. 

## 5. Conclusions

The study results provide an overview of the level of contamination in wells with Gram-negative rod-shaped bacteria and on the presence of unusual *Salmonella* serovars seen infrequently in patients but more often in reptiles and poultry. Studying animal reservoirs would provide useful information to identify the source of such contaminations. External factors leading to contamination of water have been identified. Building a well with an impervious frame, for example, might be a first step protecting water from contamination. In general, more insight into the risks of water contamination and its link to human infections are needed to promote measures effective in the reduction of water-borne disease. This is especially necessary as improved water sources are not necessarily safe water sources, given the high concentrations of Gram-negative rod-shaped bacteria and intermittent presence of *Salmonella*.
